# MYC’s Fine Line Between B Cell Development and Malignancy

**DOI:** 10.3390/cells9020523

**Published:** 2020-02-24

**Authors:** Oriol de Barrios, Ainara Meler, Maribel Parra

**Affiliations:** Lymphocyte Development and Disease Group, Josep Carreras Leukaemia Research Institute, IJC Building, Campus ICO-Germans Trias i Pujol, Ctra de Can Ruti, 08916 Barcelona, Spainameler@carrerasresearch.org (A.M.)

**Keywords:** MYC, B cell development, leukemia, lymphoma

## Abstract

The transcription factor MYC is transiently expressed during B lymphocyte development, and its correct modulation is essential in defined developmental transitions. Although temporary downregulation of MYC is essential at specific points, basal levels of expression are maintained, and its protein levels are not completely silenced until the B cell becomes fully differentiated into a plasma cell or a memory B cell. MYC has been described as a proto-oncogene that is closely involved in many cancers, including leukemia and lymphoma. Aberrant expression of MYC protein in these hematological malignancies results in an uncontrolled rate of proliferation and, thereby, a blockade of the differentiation process. MYC is not activated by mutations in the coding sequence, and, as reviewed here, its overexpression in leukemia and lymphoma is mainly caused by gene amplification, chromosomal translocations, and aberrant regulation of its transcription. This review provides a thorough overview of the role of MYC in the developmental steps of B cells, and of how it performs its essential function in an oncogenic context, highlighting the importance of appropriate MYC regulation circuitry.

## 1. The Role of MYC in B Cell Differentiation

**Figure 1:**
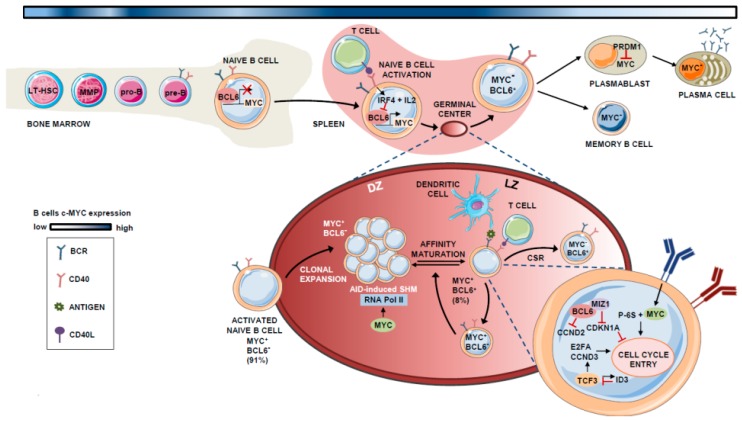
Expression and role of MYC in B lymphocyte differentiation. Schematic representation of the participation of the MYC protein throughout B-cell differentiation in the bone marrow and germinal center (GC). The percentages shown refer to the population of MYC^+^, BCL6^+/−^ cells in the total number of B cells present in the GC. The blue-colored line at the top of the Figure indicates the evolution of MYC expression, where darker blue indicates steps that require higher MYC levels.

Hematopoietic stem cells (HSCs) give rise to mature B cells through the sequential differentiation of lymphoid progenitors. Long-term HSCs (LT-HSCs) have the ability to self-renew and reconstitute the entire immune system by differentiating into short-term HSCs (ST-HSCs). ST-HSCs differentiate into multipotent progenitors (MPPs) that branch later into common myeloid progenitors (CMPs) and lymphoid-primed multipotent progenitors (LMPPs) [1]. LMPPs become common lymphoid progenitors (CLPs) [2], which have the potential to differentiate into B and T lymphocytes, as well as natural killer (NK) cells [2]. Once committed to the lymphoid lineage, additional differentiation steps lead to the formation of pro-B and pre-B cells, which are the early B cell precursors for immature and germinal center (GC) B cells. Bone marrow-escaping mature naïve B cells receiving T cell-dependent signals become activated and localize to the GCs. At this point, they undergo massive proliferation and programmed Ig mutation coupled to antibody affinity-based selection, a process triggered by somatic hypermutation (SHM) and class switch recombination (CSR). Finally, they differentiate into memory B cells or plasma cells (PCs) [3,4] (Figure 1).

The inhibition of erythroid differentiation was the first evidence of MYC activity in vitro, leading to the suggestion that it could have a role in hematopoietic cell development [5,6]. Moreover, the findings that some type of retroviruses expressing MYC provoke the formation of hematopoietic tumors, such as myeloid leukemia [7], and that its expression is deregulated in Burkitt lymphoma [8], reinforced the idea of the potential involvement of MYC in hematopoiesis. In the specific case of B lymphocytes, the use of transgenic mice overexpressing MYC revealed a developmental blockade at the B cell stage, before the onset of lymphoma [9].

Given the importance of MYC deregulation in human leukemia and lymphoma, it is not surprising that its correct modulation is essential throughout the whole B lymphocyte development [10]. At the LT-HSC stage, there is a combined expression of c-MYC and N-MYC isoforms, but there is a complete absence of L-MYC family members [11]. Interestingly, MYC expression allows LT-HSCs and MPPs to be distinguished [10]. On the one hand, LT-HSCs display low levels of MYC to maintain a tight equilibrium between cell self-renewal capacity and differentiation. On the other hand, the activation of MYC expression promotes the differentiation of LT-HSCs into MPPs, which present increased proliferating activity [10,12]. 

Despite its role in maintaining the self-renewal capacity of LT-HSCs, MYC is also essential for controlling proper hematopoiesis. In fact, *Myc*-deficient murine embryos exhibit impaired hematopoiesis and die before mid-gestation [13]. At this developmental stage, the role of MYC proteins is hierarchical. N-MYC and L-MYC cannot be expressed alone and require the concomitant expression of c-MYC. For instance, the single deletion of N-MYC does not affect the quiescent state of HSCs or hematopoiesis, whereas the deletion of c-MYC in HSCs alters proliferation and survival [11]. In summary, c-MYC is essential for balancing self-renewal and differentiation at the HSC stage. Sustained expression of MYC encompasses the transition from HSCs to lymphoid-committed cells since its extensive ability to bind to promoter and enhancer regions endows it with an extensive gene transcription role in both developmental stages [10,14].

N-MYC and c-MYC are both expressed in lymphocyte progenitors, meanwhile only the expression of c-MYC is maintained during the rest of the differentiation process, despite being reduced in precursor and mature B cell stages [15]. MYC expression is induced in pre-B cells in response to B-cell receptor (BCR) stimulation [16,17]. MYC expression peaks coincide with the stages of higher proliferative rates in B lymphocyte generation [18]. In consequence, MYC has an essential role in the expansion of pro-B cells and differentiation to the pre-B stage [10]. Conditional knockout of c-MYC or N-MYC using the *Cd19*-Cre transgenic mouse model blocks the transition from pro-B to pre-B cells, confirming its role at this stage of lineage development [19].

In connection with these data, aberrant expression of MYC in transgenic mice results in a reduction of mature B lymphocyte numbers relative to those of pre-B cells [9]. In a similar way, the regulation of MYC expression may be altered by the presence of the antiapoptotic factor BCL2 [20], or the stimulation with cytokines, such as interleukin 7 (IL-7) [21], resulting in a tumorigenic outcome, given the ability of these two proteins to enhance cell survival. Conversely, *Myc*-null B lymphocytes have an impaired proliferation capacity when treated with stimulatory cytokines, such as the B-cell surface antigen CD40 and IL-4 [22]. 

During the complex program that naïve B cells undergo in the GC before they differentiate to memory B cells or PCs, the expression of MYC is maintained, though being depleted when the B cell exits de GC reaction [15,21]. In this context, MYC is basically restricted to specific phases of the GC reaction development and is mainly expressed during naïve B cell expansion and at stages preceding the light zone (LZ) to dark zone (DZ) transition [23,24].

B-cell lymphoma 6 (BCL6) is a direct repressor of MYC during the GC reaction [23]. BCL6 binds to the promoter region of *MYC* in pre-B and differentiated B cells [25,26,27]. Therefore, the expression pattern of both factors is mutually exclusive in most GC B cells, with 91% of those cells expressing either BCL6 or MYC, and only 8% showing co-expression of both proteins [23]. In GCs, when B cells interact with antigens and access T-helper (Th) cells, they transiently express MYC due to the transcriptional inhibition of *BCL6* by the repressive machinery comprising BCR, IL-2, and interferon regulatory factor 4 (IRF4), the latter being induced upon CD40 activation [24,28,29]. In the LZ, the BCR also synergizes with CD40 to activate MYC and induce p-S6, allowing cell-cycle entry [30,31].

In these early stages of GC formation, MYC-expressing B cells express cyclin D2 (CCND2) [32,33] and D3 (CCND3) [34,35], which possibly contributes to their hyperproliferative phenotype during the initial rounds of cell division that give rise to the bulk of the GC B cells [36]. As described by Victora et al., B cell clonal expansion is restricted to the DZ, and cells move to the LZ in a bi-directional process controlled by T cells. Based on the amount of Ag captured, Th cells at the LZ determine whether MYC^+^ B cells re-enter the DZ for additional rounds of positive selection, or if they remain in the LZ [37].

MYC^+^ B cells at the LZ subsequently undergo transcription, whereby BCL6 binds the transcription factor (TF) MYC-interacting zing-finger protein 1 (MIZ1) [38], an MYC partner that acts to suppress CDK inhibitor p21 and thereby induce cell-cycle entry. At this stage, BCL6 and MYC are co-expressed in the LZ [23]. BCL6 also inhibits *CCND2* expression [32,33], which is an MYC target. CCND3, which is not controlled by MYC [34,35], is expressed alone in these LZ GC B cells. The TF TCF3 (also called E2A) is intrinsically regulated by the induction of its own inhibitor ID3 (inhibitor of DNA binding 3), is expressed in the GC B cells, and activates *CCND3* and *E2F2*, replacing CCND2-dependent proliferation in the LZ MYC^+^ B cells [27,39].

Logically, MYC expression must be tightly controlled in the DZ to limit cell divisions before each round of antigen affinity-based selection, as MYC controls the transcriptional pause release of RNA polymerase II, which is essential for activation-induced cytidine deaminase (AID)-induced somatic hypermutation (SHM) [40,41]. After several rounds of positive selection, the MYC^-^ B cells finally exit the GC and become either B memory cells or plasmablasts. B-lymphocyte-induced maturation protein 1 (BLIMP1) suppresses *MYC* expression in plasmablasts and induces PC differentiation [42]. This dependency effect between MYC and B cell proliferation is known as “cyclic re-entry” [23]. A schematic summary of the role of MYC in B lymphocyte differentiation is shown in Figure 1.

## 2. MYC Role in Leukemogenesis

Unlike other proto-oncogenes, *MYC* is not activated by oncogenic mutations in the coding sequence. MYC transforms cells via aberrant overexpression of intact MYC protein by three main mechanisms: gene amplification, chromosomal translocation, and aberrant regulation of its expression. In the following sections, we describe the role of MYC in several types of leukemia.

### 2.1. B lymphoblastic Leukemia with t(9;22) BCR-ABL1 Rearrangement

The B-cell receptor – ABL proto-oncogene 1 (BCR-ABL1) fusion (a translocation widely known as the Philadelphia chromosome, Ph) protein product can activate *Myc* in bone marrow-derived murine pre-B cells [43]. The activation of *MYC*, combined with other oncoproteins, such as RAS, c-RAF, and c-JUN, promotes the activation of signaling pathways, leading to malignant cell transformation [44]. Remarkably, the repression of *MYC* impairs BCR-ABL1-mediated transformation, indicating that MYC not only has a complementary function but also is essential for ensuring leukemic transformation [43,45].

Whereas the activation of *MYC* in lymphomas is partially caused by an elevated mutation frequency in several cases, B-cell precursor leukemia has an almost negligible mutation rate [46]. However, BCR-ABL rearranged pre-B-acute lymphoblastic leukemia (ALL) is driven by an aberrant expression of AID [47], which is expressed at such an early stage of B lymphocyte development [48], as a consequence of the enhanced kinase activity of BCR-ABL1 fusion protein (i.e., tyrosine kinase P210) [47,49]. Nevertheless, the proportion of patients harboring mutations at the *MYC* gene itself among Ph^+^ ALL cases remains low and stable compared with that of Ph^-^ patients [47]. 

In line with these data, *MYC*-*IGH* translocation, which is a common alteration in B-cell lymphomas [50], is not frequently present in the B-cell precursor ALL. However, when analyzing the genetic deletion of *CDKN2*, a common B-ALL feature, it was found that patients with the wild-type *CDKN2* experienced a higher rate of *MYC*-*IGH* translocation [51], suggesting that the two genetic alterations may be mutually exclusive.

MYC is induced through different pathways triggered by the BCR-ABL1 fusion protein. For instance, the *MYC* gene is one of the pre-BCR downstream effectors whose signaling is transduced through spleen tyrosine kinase (SYK) [52,53]. The inhibition of SYK impairs cell viability via the repressed transcription of *MYC* oncogene [53]. In parallel, the pro-inflammatory marker sphingosine kinase 2 (SK2) promotes the activation of MYC in murine models of B-ALL by increasing its acetylation profile. The inhibition of SK2 provokes a drastic reduction in ALL cell proliferation through concomitant repression of MYC target genes [54]. Recently, the use of purinostat mesylate (a first-in-class histone deacetylase (HDAC) inhibitor with reported antitumor activity [55]) has also been shown to downregulate the BCR-ABL1 fusion protein targeting of *MYC* through the alteration of global histone 3 (H3) and histone 4 (H4) acetylation [56]. These studies reveal chromatin remodeling to be a promising therapeutic strategy in BCR-ABL1^+^ ALL. For instance, as described in greater detail below, the combination of HDAC and PI3K inhibition impairs MYC-dependent growth in hematological malignancies [57].

The Wnt signaling cascade is a well-characterized oncogenic pathway that can drive *MYC* oncogene activation. The BCR-ABL1 protein phosphorylates specific tyrosine residues of γ-catenin, thereby enhancing the direct binding of this effector to the *MYC* promoter [58]. The role of this kinase activity differs from that in HSCs, where BCR-ABL1 phosphorylates β-catenin, giving rise to initial forms of chronic myeloid leukemia (CML), without requiring MYC induction [58,59]. Instead, BCR-ABL1-driven activation of the JAK/STAT pathway through the phosphorylation of JAK2 has similar effects on both chronic myeloid leukemia (CML) and ALL, whereby pJAK2 and pSTAT5 cooperate to maintain elevated levels of MYC by protecting it from ubiquitin-dependent degradation [60,61].

MYC is also regulated at several post-transcriptional levels in Ph^+^ B-ALL. Since the highly structured 5’-UTR of *MYC* determines its translation rate, eIFs are key translation factors that enable *MYC* mRNA translation [62]. IgM signaling, which is active in chronic lymphoid leukemia (CLL) cells, promotes increased translation of *MYC* mRNA, together with the induction of eIF4 and eIF4GI [63,64]. eIF4 and MYC participate in a feedforward loop that enhances both activities [65]. This is not the only mechanism through which MYC reinforces its own expression. For instance, MYC, in cooperation with its TF partner MAX, binds to the promoter of *BCR*-*ABL1*, activating its transcription [66].

The aberrantly activated function of MYC in ALL also depends on protein stabilization. Some of the first evidence demonstrated that the induction of the Ras pathway prevents proteasomal-mediated degradation of MYC [67,68]. Moreover, most leukemia cell lines harbor an altered MYC form with a prolonged half-life, without possessing genetic mutations or chromosomal alterations [47,69]. The increased stability of MYC is explained by an excess of phosphorylation at Ser62, combined with low levels of pThr58, which promote glycogen synthase kinase 3 beta GSK3β-mediated ubiquitylation and proteasomal degradation [69,70].

**Figure 2:**
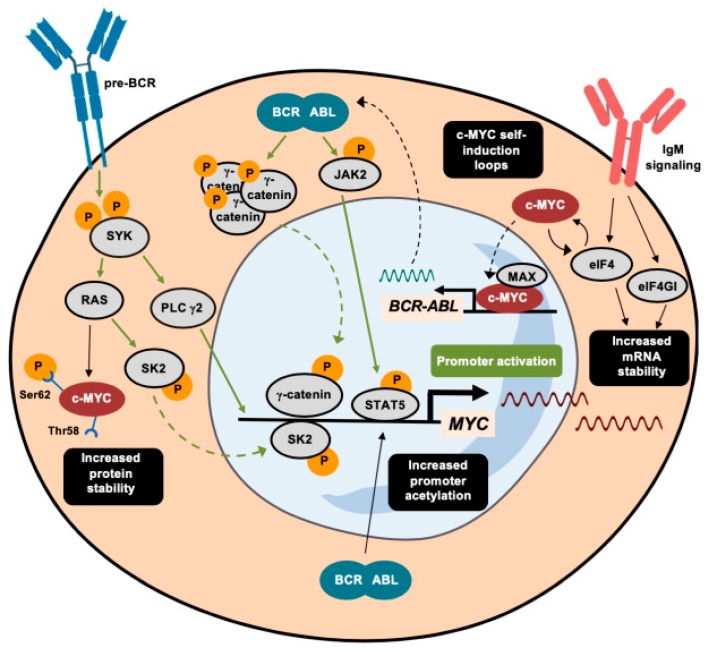
Activating mechanisms of c-MYC in leukemia with the BCR-ABL1 rearrangement. A summary of the different transduction signaling pathways that trigger the activation of MYC promoter in BCR-ABL1-rearranged leukemia. Apart from direct transcriptional activation pathways, marked in green, alternative mechanisms that induce c-MYC are depicted in black and highlighted in black squares. Dashed arrows indicate the translocation of proteins between the nucleus and the cytoplasm.

Apart from its main function in driving tumor progression, MYC also induces apoptosis, since it targets some genes involved in the BCL2 network [71,72]. As part of this network, the apoptosis-inducer protein BIM acts as a major antagonist of BCL2. In fact, the *Eµ-myc* murine leukemia model has demonstrated that the deletion of *Bim* counteracts the potential induction of cell death by MYC, worsening the B-cell leukemia-associated prognosis of these mice [73]. The regulation of BIM is partially mediated by the miR-17-92 cluster (also known as MIR17HG), in MYC-driven leukemia [74,75] and the inhibition of this specific microRNA endows leukemic cells with a pro-apoptotic phenotype [75], making microRNA networks an alternative entry point for interfering with MYC function in the B-cell precursor ALL. The regulation of MYC in BCR-ABL1-rearranged leukemia is depicted in Figure 2.

### 2.2. B lymphoblastic Leukemia with the t(v;11) MLL Rearrangement

Translocations in the histone methyltransferase MLL gene are the most common chromosomal alteration in infant leukemia and, in general, exhibit a very poor prognosis, such that the disease is an extremely lethal malignancy in infants [76,77,78]. As in other types of B-cell leukemia, the *MYC* gene is not commonly involved in chromosomal translocations, although rare individual cases with t(8;22) have been reported [77]. Recently, a revised characterization of the RS4;11 leukemic cell line has demonstrated the presence of i(8q), resulting in *MYC* duplication, which confers a selective growth advantage in vitro [79]. In B-ALL, this alteration is considered a secondary hit that contributes to disease progression. Patients with the MLL-AF4 fusion protein have a strongly enriched *MYC* gene signature compared with AML patients [80,81].

Consequently, MLL-fusion proteins activate the expression of the *MYC* oncogene in pre-B and pro-B-cell leukemia [82,83]. For instance, MLL protein can prompt additional activity by fusing to USP2 deubiquitinating protein, leading to the enrichment of USP2 activity on MDM2, ending with the enhanced degradation of p53, which, in turn, activates MYC expression [84,85].

The binding of MLL-rearranged proteins to the regulatory regions of target genes depends on the presence of chromatin adaptors that comprise the super-elongation complex (SEC). The bromodomain and extra-terminal domain (BET) family of proteins (BRD2/3/4) are part of this complex and contribute to the induction of MYC [86,87,88]. Initial evidence showed that suppression of BRD4 induces potent cell growth arrest and cell senescence, combined with MYC downregulation, meaning that the BET family is a promising therapeutic target [86,87,88,89].

For instance, a small molecule inhibitor of BET (iBET-151) prevents the recruitment of BET proteins to chromatin by inhibiting the transcription of key targets, such as *BCL2* and *MYC* [87]. In parallel, JQ1 (a potent inhibitor of BRD4) greatly reduces MYC expression and activity, jointly with a large set of its target genes [88]. Moreover, JQ1 has also been tested in patient-derived xenograft from ALL patients, confirming its ability to inhibit MYC expression [90]. BET proteins are involved in maintaining aberrantly altered chromatin states in ALL. At this point, BRD4 cooperates with MYC in recruiting TEFb complexes to initiate transcriptional elongation at active promoters, while the transcriptional regulator HEXIM1 counteracts BRD4 and MYC role by inactivating the complex. Therefore, tight regulation of BRD4, MYC, and HEXIM1 is required for proper elongation [40,91,92]. A novel oral BRD2/3/4 inhibitor (OTX015) has been shown to reduce MYC expression and to increase HEXIM1 levels in MLL-rearranged leukemia [89]. Apart from OTX015, the specific BRD4 inhibitor CPI-0610 has been selected for phase I clinical trials in ALL patients [93].

Histone deacetylases (HDACs) have a differential expression pattern in ALL patients with MLL rearrangement and are commonly overexpressed. For instance, HDAC9 is associated with an adverse prognosis, whereas SIRT1 is involved in drug resistance through its regulation of the acetylation of the *TP53*, *MYC,* and *NF*-*κβ* genes. Consequently, HDAC inhibitors (HDACis) have emerged as potential therapeutic options in treating hematological malignancies [94,95]. However, conceptualizing the role of HDACs in leukemia as inducers of malignant transformation would be a too simplistic view, given that some histone deacetylases, such as HDAC7, carry out an opposite function. As reported by Barneda-Zahonero et al., HDAC7 is involved in the repressive transcriptional machinery of MYC and, therefore, it is often reduced in different types of leukemia and lymphoma, including MLL-rearranged malignancies [96].

In this sense, newly developed compounds that selectively inhibit specific HDAC subtypes are gaining relevance in the treatment of hematological malignancies [97]. For instance, class I/IIb-selective HDACi purinostat has demonstrated a direct effect on MYC downregulation [56], while other selective drugs (mocetinostat, entinostat) are already undergoing clinical trials for diverse hematological malignancies [97]. The use of combinatorial therapies merging selective HDACi and classical treatments emerges as a promising therapeutic option, and it is tempting to speculate that its ability to induce apoptosis resides, at least partially, in MYC negative regulation [97].

The relevance of the *MYC* oncogene in hematopoiesis is restricted to its functions in aberrantly proliferating B-cell precursors and in the normal hematopoietic stem cell hierarchy. The expression of MYC throughout this process is controlled by a super-enhancer region located 1.7 Mb downstream of the gene [98]. This super-enhancer, known as the “blood enhancer cluster” (BENC), is comprised of several selectively active modules that recruit a wide range of transcription factors due to the increased chromatin accessibility. This differential access to regulatory regions has also been reported in murine models of MLL-AF9-driven leukemia, indicating that *MYC* hyperactivation during leukemia can be driven by BENC-unbalanced modulation [99,100]. BENC deletion entails a drastic depletion of B lymphocytes during normal development, as well as an improved prognosis in MLL-AF9^+^ leukemia [100]. Regarding the regulation of *MYC* at the promoter level, we strongly consider that recently developed techniques for 3D chromatin architecture analysis will improve our knowledge about the coordination of transcriptional machinery, chromatin accessibility, and 3D structure. Not in vain, this novel methodology has already conferred a new dimension to the study of B cell development at different stages [101].

### 2.3. B Lymphoblastic Leukemia with the t(12;21) ETV6/RUNX1 Rearrangement

The t(12;21) translocation, which involves the *ETV6* and *RUNX1* (also known as AML1) genes, is the most frequent lesion in childhood B-ALL (20–30% of cases), at early diagnosis and remission [102,103]. The N-terminal region of ETV6 displays weak homology with the bHLH region of MYC protein [104]. This homology enables the induction of the targets of these factors through protein-protein interaction, enhancing MYC oncogenic function [103]. Apart from its characteristic fusion to RUNX1 protein, ETV6 also forms fusion proteins with PAX5, which is a key inducer of B-cell-specific genes (such as *CD19* and *CD79A*) [104,105]. The combination of PAX5 activity with ETV6-mediated MYC targets induction establishes the ETV6/PAX5 fusion protein as a powerful mediator of ALL progression.

Alterations affecting the *MYC* gene itself should be highlighted as examples of chromosomal aberrations. For instance, a double *MYC* gene translocation t(8;14)t(8;9) was reported in a B-ALL patient with *ETV6* amplification [106]. Copy number variation (CNV) was reported in a substantial 65% of relapsing ETV6/RUNX1-positive ALL patients, including MYC expression gain at chromosome 8 (q23.1-24.1) in 10% of cases [107].

An indirect pathway of MYC activation in ETV6/RUNX1-rearranged leukemia is mediated by the GTP-binding protein RAC1, a pivotal modulator of hematopoiesis [108], that increases the phosphorylation levels of STAT3 [109]. ETV6/RUNX1 protein enhances the activity of RAC1, increasing MYC expression, induced by the phosphorylation of STAT3 [110]. Specific STAT3 inhibitors revert MYC induction by blocking cell proliferation and promoting apoptosis in pro-B-ALL cells [110].

Additionally, the ETV6/RUNX1 fusion gene can be stabilized at the mRNA level by the RNA-binding protein IGF2BP1, which is overexpressed in this type of leukemia [111]. IGF2BP1 leads to an eventual increase of MYC, linked to aberrant leukemogenesis in ETV6/RUNX1-mediated ALL [111,112]. Finally, and similarly to the mechanism reported for BCR-ABL1-rearranged leukemia, MYC is also stabilized at the protein level through aberrantly altered phosphorylation at the Thr58 and Ser62 residues [69].

### 2.4. B Lymphoblastic Leukemia with other Chromosomal Rearrangements

Expression of B220 and CD43 determines the transition of pro-B into pre-B lymphocytes [113]. Leukemia derived from this developmental stage usually displays *TCF3*/*PBX1* chromosomal rearrangement, which is commonly found in leukemia derived from pre-B lymphocytes (in more than 90% of cases) [114]. Survival of TCF3/PBX1^+^ cells critically depends on the activity of the pre-BCR [52,53]. Immunoglobulin µ (Igµ) heavy-chain knockdown impairs the proper assembly of pre-BCR and blocks signal transduction through the Igα-Igβ heterodimer [115]. Igµ downregulation in TCF3/PBX1-rearranged cell lines significantly suppresses MYC expression at the mRNA and protein levels. MYC is regulated by the pre-BCR in a FOXO-dependent manner since the forced expression of a constitutive form of FOXO1 reverts the blockade of pre-BCR signaling, and partially restores MYC expression [53].

Under physiological conditions, *MYC* mRNA is modulated by miR-24, which is able to bind at its 3′-UTR region to reduce MYC levels, thereby controlling cell-cycle progression [116]. miR-24 is frequently downregulated in TCF3/PBX1^+^ pre-B-ALL, concomitantly with other miRNAs involved in proliferation and apoptosis modulation in various cancers (e.g., miR-126 and miR-365) [117]. Surprisingly, the restoration of miR-24 expression in *TCF3*-rearranged leukemic cell lines neither affects the expression of some of its targets nor alters the frequency of apoptotic cells, suggesting that MYC is regulated by a combination of mechanisms in this type of leukemia [117].

Despite not being the most frequent alteration, the *IGH* gene (located at chromosome 5) can also be translocated to chromosomes 14 or 12, as is the case for the NALM-6 cell line, which harbors the t(5;12) translocation. This cell line was recently used to identify the transcriptional cofactor apoptosis antagonizing transcription factor (AATF) as being a direct target of MYC since it features canonical binding motifs at the promoter region [118]. AATF promotes cell-cycle progression by inhibiting TP53 expression and mediating the response to DNA damage [119]. It is of particular note that, when inhibiting the expression of MYC, there is a drastic downregulation of *MLL* gene expression, a previously described key mediator of pediatric leukemia. This downregulation can be counteracted by the exogenous induction of AATF. Therefore, AATF mediates a positive feedback loop between MYC and *MLL* gene in pro-B-ALL [118].

## 3. MYC Role in Lymphomagenesis

In hematopoietic malignancies, genomic abnormalities involving the *MYC* gene are almost always found in B cell lymphomas, but rarely in T cell lymphomas. 30% of all lymphoid neoplasms are B cell non-Hodgkin lymphomas. These can be classified further as Burkitt lymphoma (BL), diffuse large B cell lymphoma (DLBCL), follicular lymphoma (FL), mantle cell lymphoma (MCL), and plasmablastic lymphoma (PBL), among others.

### 3.1. MYC in Burkitt Lymphomas

BL arises mostly in children and young adults and has an extremely high proliferation rate. Endemic, sporadic, and immunodeficiency-associated BLs are distinguished as clinical variants in the World Health Organization (WHO) classification. BL has a mature B cell phenotype with expression of GC/post-GC markers such as immunoglobulin M (IgM), CD10, and is typically negative for BCL2 [120].

The genetic hallmark that characterizes BL is the rearrangement of *MYC* with one of the *IG* gene loci. Translocation triggers constitutive *MYC* hypermutation of the translocated gene in germinal centers [121], subjected to AID-dependent SHM [41], which is susceptible to generating *MYC* variants and increasing its oncogenic potential [122]. Specifically, an *MYC* translocation to the *IG* heavy chain gene locus 14q32 (80% of the cases), or to the *IGκ* or *IGλ* light chain genes at 2p12 or 22q11 (10%) are the main rearrangement sites [123,124]. Most mutations of the rearranged *MYC* gene are point SNPs or deletions in the 3’ border of the first exon and the first intron [125,126], altering the coding sequence but permitting its transcription from the translocated chromosome. In consequence, there is an MYC expression that terminates inhibiting cell differentiation and inducing proliferation, probably keeping the cells in a hyperproliferative state.

In a gene expression profile study of human samples of BL (and DLBCL), three main cytogenetic groups within the mature aggressive B cell lymphomas were distinguished: MYC-simple, with IG-MYC fusions and a low chromosomal complexity score, no IGH-BCL2 fusions, and no *BCL6* breakpoints, and with a favorable prognosis; MYC-complex, including *IG/MYC*-rearranged BLs with highly complex karyotypes, non-*IG/MYC*-rearranged cases, and all IGH/BCL2 fusions and/or *BCL6* breakpoints, or any combination of these; and MYC-negative, comprising lymphomas with unaltered *MYC* [127].

Inhibitor of DNA binding (ID) proteins, such as ID3, bind E-proteins such as TCF3 via HLH common motifs, preventing the binding of the latter to DNA. Schmitz et al. shed light on some oncogenic pathways, suggesting that *MYC* translocation is insufficient to induce BL [128]. The next-generation sequencing (NGS) study performed by Love et al. identified *MYC* and *ID3* as the genes most frequently mutated in BL [128,129].

In the setting of deregulated MYC, samples with *ID3* mutations show a higher level of expression of known MYC target genes, and give rise to increased G1-to-S-phase cell-cycle progression in BL, suggesting a role for ID3 as a tumor suppressor in this type of lymphoma [129]. The high level of ID3 expression in BL might be because the *ID3* locus itself is a direct target of MYC [130] and due to BCR triggering, as described using an *Id3*^-/-^ mouse model [131]. Functional analyses suggested that *ID3*-inactivating mutations and *TCF3*-activating mutations (by blocking ID3 binding sites) lead to the activation of a TCF3-dependent transcriptional program that consequently promotes tonic BCR signaling [132]. TCF3 would repress *PTPN6*, which encodes SHP-1, a BCR-attenuating factor that acts by dephosphorylating the ITAM motifs of the CD79A and CD79B signaling subunits of the BCR [39]. In addition, BCR can activate both MYC and ID3 by a sequential process in which MYC rapidly upregulates its expression. Later, upon MYC downregulation, levels of ID3 increase [131]. This effect may be produced by direct BCR activation, or through an indirect effect of MYC, highlighting the existence of a positive feedback loop between BCR, ID3, and MYC regulation.

Tonic BCR activation requires PI3K signaling in mature B cells to maintain its continuity [132], and the pro-survival pathway cooperates with MYC in BL [133]. MYC deregulation induces the expression of the MIR17HG, a microRNA host gene amplified in ~10% of BL cases [134]. Particularly, miR-19 is the key oncogenic component of the cluster, which antagonizes PTEN and, consequently, activates the AKT-mTOR pathway, the consequence of which is exacerbated cell survival in MYC-driven lymphomagenesis [128,135,136]. See [137] for an extensive review of the involvement of MYC and miRNAs in lymphomagenesis.

BCR-induced PI3K pathway activation in BL contrasts with the absence of NF-κβ survival pathway signaling in these tumors [138]. Reinforcing this, the study by Klapproth et al. in *Myc* transgenic mice showed that constitutive NF-κβ activity is incompatible with the development of the MYC-induced lymphomas [139]. The resting state of the NF-κβ apoptotic pathway confers a selective advantage on MYC-driven oncogenic cells.

### 3.2. MYC in Diffuse Large B Cell Lymphoma

DLBCL accounts for approximately 40% of all non-Hodgkin lymphomas [120]. Two major subtypes can be identified: germinal center B-cell-like (GCB), which has a gene expression profile similar to that of the GC B cell; and activated B cell-like (ABC), which has a worse outcome because it expresses genes present in activated peripheral B cells [120]. The principal translocated sites in DLBCL are a rearrangement of *BCL6* (30% of cases) and t(14;18)(q32;q21) with *BCL2* rearrangement to the *IGH* gene locus (20–30% of cases) [140,141]. Globally *BCL2* is rearranged in 30% of cases in the GCB-DLBCL subgroup and in <5% of ABC-DLBCL cases [140,142,143].

Considering MYC specifically, its protein expression is detected in ~40% of diagnoses, but its rearrangement is found in only around 10% of them, suggesting that alternative mechanisms may be associated with MYC deregulation [143,144,145,146]. With regard to translocations, similar to what is observed in BLs, the *IG* genes are the most frequent *MYC* partners, the latter being most commonly fused to *IGH* or to non-IG genes such as *BCL6*, *BCL2*, *PAX5*, or *IKAROS*, which appear as translocation partners in 35–50% of *MYC*-rearranged DLBCLs [145,146,147]. In addition to *MYC* translocations, DLBCLs are characterized by the presence of *MYC* amplification and gains, and increased copy numbers of *MYC* are associated with higher levels of mRNA and protein, resulting in a very poor prognosis [148,149]. However, careful examination of Cosmic, a public catalog of somatic mutations in cancer, revealed that SNPs in *MYC* sequence could be detected in a significant fraction of DLBCLs, as reported in BL. According to this data and as described before, polymorphisms in *MYC* sequence do not impair its transcription, permitting in consequence, a dysregulated gene expression that could maintain the cell in a hyperproliferative state that, in the end, will endow cells with increased aggressiveness.

*MYC* rearrangements are often involved in complex karyotypes and are frequently associated with other oncogenic abnormalities. Lymphomas that carry *MYC* and either a *BCL2* or a *BCL6* translocation (a double-hit lymphoma, DHL) or all three rearrangements (a triple-hit lymphoma, THL) are included in the current WHO classification as a new entity termed “High-grade B cell lymphomas with *MYC* and *BCL2* and *BCL6* rearrangements” [150]. Molecularly, DHL with *MYC* and *BCL2* rearrangements present a *TP53* mutation, inhibiting TP53-mediated apoptosis at a higher frequency than DHL, including *MYC* and *BCL6* alterations, which account for 35% and 6% of cases, respectively [151,152]. Therefore, in the first group, upregulated MYC expression promotes proliferation and disables the capacity to induce apoptosis, while BCL2 expression fosters cell survival. Together their co-expression confers an aggressive proliferating phenotype on these DHLs. 

MYC overexpression is a reliable biomarker for predicting therapeutic response, since its expression is a poor prognostic factor in DLBCL [153] but, beyond the aforementioned rearrangements, the mechanisms underlying its overexpression are still unknown. The stability of MYC is regulated by GSK-3β, which phosphorylates MYC at Thr58 and induces its degradation via the ubiquitin-proteasome pathway [154]. Wang et al. demonstrated that BCR stimulation could activate downstream PI3K signaling, phosphorylating GSK-3β at Ser9, and abolishing its ability to induce MYC degradation in DLBCL [155]. Moreover, the PI3K pathway inhibitory elements such as PTEN are frequently lost in GCB-DLBCL [156], while BCR mutations also result in its constitutive activation [157], leading to MYC dysregulation in DLBCL.

Finally, the upregulation of MYC expression in DLBCL promotes BCR signaling by inducing the MIR17HG cluster, employing a mechanism similar to that described above in the section on BL [158,159]. Taken together, these data suggest that a positive feedback loop operates in the BCR-PI3K-MYC signaling axis in DLBCL.

### 3.3. MYC In Plasmablastic Lymphoma

PBL is an aggressive, high-grade lymphoma that is most commonly diagnosed in patients with HIV infection or an immunocompromised phenotype [120]. The cell of origin in PBL is thought to be the plasmablast, an activated B cell that has undergone SHM and CSR, and that expresses cell surface markers such as CD138, CD38, MUM1, and Ig, similar to a plasma cell [120]. Signaling pathways leading to plasma cell differentiation involve gene silencing of *PAX5* and *BCL6* through BLIMP1 [160,161], which also represses *MYC* expression through promoter binding [42]. Recurrent somatic mutations in *PRDM1* (the gene encoding BLIMP1) occur in 50% of cases, where they affect the regulation of diverse targets, such as *MYC* [162]. Moreover, MYC and BLIMP1 proteins were found to be co-expressed in 80% of diagnoses [162]. These findings are firm evidence that PRDM1 contributes to the oncogenicity of dysregulated MYC.

Although *MYC* rearrangement is the genetic hallmark of BL and is characteristic of an aggressive subset of DLBCL, it is also a common finding in PBL, along with *MYC* gains [163,164]. In PBL, *MYC* rearrangements have been found in ~50% of cases, and the *IG* genes are the most frequent partners (~85%), with t(8;14) *MYC*/*IGH* being the commonest fusion product [163]. Gene expression analysis of PBL revealed MYC overexpression at mRNA and protein levels [165]. MYC overexpression facilitates PBL cell apoptosis escape through cell-cycle dysregulation, and jointly with loss of TP53 [166]. Together, these two processes enhance the aggressiveness of PBL.

In the absence of translocations, the mechanisms of *MYC* dysregulation are poorly understood, suggesting that MYC may be activated by other mutated genes. Rearrangements of *BCL2*, *BCL6*, *MALT1*, and *PAX5*, which are common in BL and DLBCL, are not detected in PBL. Conversely, gains of these loci are frequent in PBL, where 30% of cases display amplification of three or more of them [163]. Paradoxically, Ouansafi et al. demonstrated in a single case report the concomitant presence of *BCL2* and *MYC* translocation in a rare case of FL-to-PBL transformation [167].

### 3.4. MYC in Other Non-Hodgkin B Cell Lymphomas

FL is an indolent non-Hodgkin lymphoma that transforms into a high-grade lymphoma, mostly DLBCL, in about one-third of patients. The genetic hallmark of FL is t(14;18)(q32;q21), which brings about BCL2/IGH fusion protein [168]. Low-grade lymphomas containing a *BCL2* rearrangement need subsequent secondary genetic hits for the disease to evolve. The genetic alteration of *MYC* may suffice as this secondary alteration, leading to the transformation into a high-grade B cell lymphoma [169]. Actually, the majority of FLs express MYC, but only in a small fraction of the cells (<25%) [146].

Pasqualucci et al. investigated the genetic drivers of transformed follicular lymphoma (t-FL) and determined that there is a common mutated precursor that experiences distinct genetic events that are specifically associated with alterations deregulating cell-cycle progression and DNA damage [46], evidence that matches perfectly with *MYC* oncogene among others. t-FL to DLBCL progression occurs in 30% of the cases, mainly among GCB-DLBCL patients [170,171]. t-FL oncogenic mechanisms are characterized by the presence of a proliferation signature, together with recurrent oncogenic transformations such as *TP53* mutation, *CDKN2A* loss, and c-*REL* amplification [171], giving rise to a proliferative phenotype in which MYC could be involved. In fact, genetic lesions deregulating *MYC* are the second most common tFL-specific lesion (including translocations, point mutations, and CNVs) [46]. Alternative pathways involving MYC and its targets could help distinguish between two types of morphologically similar lymphomas, such as tFL-derived DLBCL and de novo DLBCL, this signature being more enriched in de novo cases than in transformed ones [172].

As reported by Martinez-Climent et al., when examining gene expression changes in t-FL, a considerable number of MYC target genes are differentially expressed, although the *MYC* gene locus remains unaltered in terms of copy number [173]. Consequently, *MYC* genetic abnormalities are not the driving mutations of FL transformation and may only serve as a surrogate for the entire proliferation signature.

MCL is generally an aggressive malignancy, but it is thought in some cases to remain latently quarrelsome in an indolent phase. It is characterized by t(11;14)(q13;q32), juxtaposing *IGH*, and *CCND1*, resulting in CCND1 overexpression, which drives the cells through the G1/S transition [174]. Interestingly, a partnership between CCND1 and MYC has been reported in the oncogenic transformation of B cells to MCL [175]. The coexistence of *MYC* and *CCND1*/*IGH* rearrangements [176] is commonly found in double-hit (DH)-MCL [177], which is associated with a high-risk prognostic index. As reported for FL, most of the MCL cases display an intense MYC expression, but the percentage of positive cells is frequently low (<25%) [146].

Aggressive MCL subset variants can be divided between the blastoid variants (resembling lymphoblast cells) and pleomorphic variants (DLBCL-like cells). MCL is also characterized by large numbers of secondary gains and losses of genes that are mainly involved in cell-cycle regulation, response to DNA damage, and survival [178]. Regarding the blastoid variant, *MYC* alterations such as the rearrangement involving *IGH* in t(8;14), disruption of the *MYC* locus in t(2;8), and gains in add(8)(q24) have been described [179,180]. These aberrations, along with a high level of TP53 expression, are features associated with MCL aggressiveness [181].

CDK4 and CDK6 are catalytic subunits of the cyclin D family that govern G1-to-S-phase progression; p16^INK4a^ and all members of the INK4 family act as their negative regulators by specifically binding to them [182]. *CDK4* mutations abolish the binding motif of the INK4 family, thereby functioning as an oncogene capable of directing proliferation [183]. Surprisingly, cyclin D1/CDK4 and p16^INK4a^ complexes are known to be upstream regulators of MYC [184], while *CDK4* has been identified as a target of MYC [185]. In the context of MYC dysregulation, CDK4-INK4 imbalance plays a role in lymphomagenesis [183].

The MYC-driven gene expression network is maintained through the stability of the MYC protein, which itself is sustained in MCL by MALT1 [186]. Constitutively activated MALT1 expression, together with BCL10, is orchestrated by the activation of the BCR, which recruits CARD11 scaffold protein and ultimately results in a BCR-driven CARD11-BCL10-MALT1 (CBM) complex. CBM subsequently activates the NF-κβ pathway [187] and, combined with MYC stabilization by MALT1, drives lymphomagenesis progression.

Table 1 summarizes the essential information about the aberrant activation of MYC in leukemia and lymphoma disorders (Chapters 2 and 3).

## 4. Concluding Remarks

The appropriate path that lymphoid progenitors should ideally follow on their way towards fully differentiated B cells is constantly under threat of being wrecked by the alteration of regulatory mechanisms. Beyond its widely known function as an oncogene, MYC also plays an essential role at different steps of B-cell differentiation, and its deregulation is one of the main hazards that can disrupt the process. As described in this review and in a physiological context, MYC is strongly expressed on the way to producing mature B lymphocytes, whereas its transient downregulation is required at some specific points. However, MYC basal levels are maintained and are not completely switched off at any point before the late memory and plasmatic B cell stages, demonstrating that only tight regulation of MYC levels ensures that the B lymphocytes achieve their correct fate.

When the expression of MYC protein is aberrantly altered, the risk of developing a hematological malignancy, such as leukemia or lymphoma, increases substantially through the acquisition of an uncontrolled proliferative rate and a blockade of differentiation. Remarkably, the alterations that trigger MYC overexpression differ between leukemia and lymphoma cells. In fact, leukemic cells have low rates of *MYC* mutations and a low frequency of chromosomal translocations involving the *MYC* gene, whereas the aforementioned genetic alterations are a hallmark in some types of non-Hodgkin B cell lymphoma. Nevertheless, and even when not altered at the genetic level, the expression of MYC is usually disrupted in the commonest types of leukemia, where it is activated by several pathways, as well as at the post-transcriptional level. Most types of lymphoma present high levels of MYC expression that are not always correlated with a mutated *MYC* gene, opening the door to speculation that, as in leukemia, multiple pathways may act to facilitate its dysregulation.

In terms of therapeutic perspectives, the possibilities for interfering with MYC activity are still to be adequately explored. However, the identification of regulatory cascades and other mechanisms that trigger its induction opens a wide range of possibilities for indirectly impairing MYC function. As reported here, we believe that the disruption of altered epigenetic regulation with HDAC inhibitors, the blockade of microRNAs function, or the use of BET inhibitors that obstruct scaffold transcriptional activating machinery, are but a few examples of the promising therapeutic strategies that will lead to an improved prognosis of hematological disorders, mostly mediated by the maintenance of MYC at physiological levels.

## Figures and Tables

**Table 1:** MYC alterations in leukemia and lymphoma. Summary of the gene alterations, chromosomal translocations, regulatory pathways, and post-transcriptional modifications involved in MYC activation that are included in this review, assigned to their corresponding subtype of leukemia and lymphoma.

		Gene Alterations (Mutations, CNV)	Involvement of Translocations	Activating Pathways	Myc Stabilization at Post-Transcriptional Level
LEUKEMIA	t(9;22) *BCR/ABL1* rearrangement	Almost negligible mutation rate [46]	*MYC*/*IGH* translocation is uncommon [50]. Higher frequency in *CDKN2* WT patients [51]	Induced by aberrant AID [47], pre-BCR/SYK signaling [53], Wnt [58] and JAK/STAT [60,61] pathways	Translation rate controlled by EIFs [62,63,64,65], prevention of proteasomal degradation [67,68,69], induction mediated by MIR17HG cluster [74,75]
	t(v;11) *KMT2A* rearrangement	i(8q) *MYC* duplication in RS4;11 cell line [79]	*MYC* is not commonly involved in translocations. Only rare cases with t(8;22) reported [77]	Indirect activation through TP53 inhibition [84,85], direct binding of MLL-fusion proteins and BET adaptors [86,87,88], regulation by BENC super-enhancer region [99,100]	
	t(12;21) *ETV6/RUNX1* rearrangement	*MYC* gene expression gain 8(q23.1-24.1). CNVs reported in 65% of cases [107]	Double *MYC* gene translocation reported [106]	ETV6 also fuses to PAX5 and induces MYC expression [104]. Also triggered by RAC1-STAT3 [110]	Stabilized at mRNA level by IGF2BP1 [111] and at the protein level by prevention of proteasomal degradation [69]
LYMPHOMA	Burkitt lymphoma	Point SNPs and deletions in the 3’border [125,126]	*MYC/IGH* t(8;14q32) in 80% of the cases and *MYC/IGκ* or *Igλ* t(8;2p12) or t(8;22q11) in 10% of the diagnoses [123,124]	BCR-induced PI3K pathway in cooperation with TCF3, ID3 and SHP-1 [129,130,131,132,133,134]	
	Diffuse large B cell Lymphoma	Point SNPs, amplifications, gains and increased copy numbers of *MYC* [148,149]	Found in 10% of the cases, being *MYC/IGL* the most common [144], but also to *BCL6*, *BCL2*, *PAX5*, *IKAROS* [145,146,147]. Often participate in complex karyotypes and associatedo a second hit such as *MYC/BCL2* and *MYC/BCL6* [151,152]	Mutations in the BCR or PI3K pathway inhibitory elements [156,157]	GSK-3β phosphorylation abolishing MYC degradation [155] MYC upregulation promotes BCR signaling by induction of MIR17HG cluster [158,159]
	Plasmablastic lymphoma	Gains [163,164] and somatic mutations in MYC inhibitor PRDM1 [42,162]	Observed in 50% of the cases, being *MYC*/*IGH* the most common [163]		
	Follicular lymphoma	Remains unaltered in terms of copy number [173]	Second most common tFL-specific lesion [46]	Proliferation signature with oncogenic transformations such as *TP53* mutations, *CDKN2A* loss and *c-REL* amplification [171]	
	Mantle cell lymphoma	Disruption of *MYC* locus in t(2;8) and *MYC* gains at (8)(q24) [179,180]	Coexistence of *CCND1/IGH* and *MYC* rearrangements [176,177], and also *MYC/IGH* t(8;14) [179,180]	CCND1/CDK4 and p16^INK4a^ imbalance [183,184,185]	BCR-driven CARD11-BCL10-MALT1 complex together with Nf-κβ pathway orchestrates MYC stability [186,187]
